# Efficacy and Safety of Single-Session Endoscopic Stone Removal for Acute Cholangitis Associated with Choledocholithiasis

**DOI:** 10.1155/2018/3145107

**Published:** 2018-08-08

**Authors:** Junya Sato, Kazunari Nakahara, Ryo Morita, Nozomi Morita, Keigo Suetani, Yosuke Michikawa, Shinjiro Kobayashi, Fumio Itoh

**Affiliations:** ^1^Department of Gastroenterology and Hepatology, St. Marianna University School of Medicine, 2-16-1 Sugao, Miyamae-ku, Kawasaki 216-8511, Japan; ^2^Department of Gastroenterological and General Surgery, St. Marianna University School of Medicine, 2-16-1 Sugao, Miyamae-ku, Kawasaki 216-8511, Japan

## Abstract

**Background/Aims:**

In early endoscopic retrograde cholangiopancreatography (ERCP) for acute cholangitis due to choledocholithiasis, it is unclear that single-session stone removal can be safely performed. We examined the efficacy and safety of early single-session stone removal for mild-to-moderate acute cholangitis associated with choledocholithiasis.

**Methods:**

Among patients with mild-to-moderate acute cholangitis associated with choledocholithiasis who underwent early ERCP (n = 167), we retrospectively compared the removal group (patients who underwent single-session stone removal; n = 78) with the drainage group (patients who underwent biliary drainage alone; n = 89) and examined the effectiveness and safety of single-session stone removal by early ERCP.

**Results:**

The patients in the removal group had significantly fewer and smaller stones compared with those in the drainage group. The single-session complete stone removal rate was 85.9% in the removal group. The complication rate in early ERCP was 11.5% in the removal group and 10.1% in the drainage group, with no significant difference (P = 0.963). On comparing patients who underwent early endoscopic sphincterotomy (EST) with those who underwent elective EST after cholangitis had improved, the post-EST bleeding rates were 6.8% and 2.7%, respectively, with no significant difference (P = 0.600). The mean duration of hospitalization was 11.9 days for the removal group and 19.9 days for the drainage group, indicating a shorter stay for the removal group (P < 0.001). In multiple linear regression analysis, stone removal in early ERCP, number of stones, and C-reactive protein level were significant predictors of hospitalization period.

**Conclusions:**

Single-session stone removal for mild-to-moderate acute cholangitis can be safely performed. It is useful from the perspective of shorter hospital stay.

## 1. Introduction

According to the Tokyo Guidelines 2013 (TG13) for acute cholangitis, endoscopic transpapillary drainage is the most recommended for acute cholangitis [1, 2]. It is recommended that the timing to perform biliary drainage should be determined in accordance with the severity of acute cholangitis [3–5], with urgent drainage indicated for severe cases and early drainage for moderate cases; biliary drainage is needed when initial treatment such as antibiotic treatment is ineffective for mild cases. Moreover, when performing early endoscopic retrograde cholangiopancreatography (ERCP) for acute cholangitis associated with choledocholithiasis, if the cholangitis is severe, it is recommended to perform biliary drainage alone [3]; however, in moderate-to-mild cases, there is insufficient evidence regarding whether or not single-session stone removal can be safely performed.

Some previous reports indicate that endoscopic sphincterotomy (EST) for patients with acute cholangitis has a risk of bleeding [6, 7] and that acute cholangitis in itself is a risk factor for post-EST bleeding [8]. Furthermore, in acute cholangitis, stone removal may carry the risk of exacerbating cholangitis. On the other hand, if the acute cholangitis is mild-to-moderate, when performing early ERCP, it has been reported that EST does not increase the bleeding rate and that it shortens the length of hospital stay [9]. In a single-arm prospective study, it was reported that, in the absence of coagulopathy, single-session stone removal by EST for mild-to-moderate acute cholangitis is effective and can be safely performed [10]. In another report [11] comparing the therapeutic outcomes of single-session stone removal with elective stone removal after drainage for acute cholangitis, there was no difference in the rates of cholangitis improvement and procedural complications. However, the report [11] had limitations such as a small number of cases and a low single-session stone removal rate of 30.5%.

In the present study, we retrospectively examined patients who underwent early ERCP for mild-to-moderate acute cholangitis associated with choledocholithiasis and compared the therapeutic outcomes and safety of single-session stone removal with biliary drainage alone.

## 2. Materials and Methods

### 2.1. Patients

During the period from January 2011 to March 2017, 212 consecutive patients with mild-to-moderate acute cholangitis associated with choledocholithiasis underwent early ERCP at the Department of Gastroenterology and Hepatology of St. Marianna University School of Medicine. Early ERCP was defined as ERCP performed within 24 h of diagnosis. Among these patients, 14 who underwent postoperative reconstruction other than the Billroth I reconstruction, 21 with concurrent gallstone pancreatitis, and 10 with concurrent cholecystitis were excluded; thus, a total of 167 patients were included in the study. Six of 167 patients were enrolled twice because of recurrence of choledocholithiasis and cholangitis during the study period. For those six patients, the time to recurrence was 13.2±7.5 months (mean±standard deviation), and complete stone removal was confirmed by cholangiography using a balloon catheter at the initial ERCP.

In early ERCP, stone removal was attempted in a single session for 78 patients (removal group) and biliary drainage alone was performed in 89 patients (drainage group). Six patients registered twice were enrolled in 7 in the removal group and 5 in the drainage group. The decision to perform single-session stone removal or drainage alone was made at the discretion of the endoscopist; however, patients receiving antithrombotic therapy for native papilla underwent drainage alone. We discontinued any kind of antithrombotic drugs in principle and EST was performed after the appropriate discontinuation period for each drug. For the patients who had difficulty in discontinuation of antithrombotic drugs, heparin was substituted. All ERCPs were performed under the supervision of an expert with experience in over 500 ERCP procedures. In our institution, biliary cannulation is first attempted using the conventional contrast cannulation (CC). However, in case where biliary cannulation is difficult to perform by CC but in which a guidewire can be placed in the pancreatic duct, the pancreatic duct guidewire placement method (P-GW) is employed as the second choice. Then, we usually place a pancreatic stent for the prevention of pancreatitis over the guidewire used in the P-GW. The high-frequency device used in EST was 120-Watt Endocut mode Effect 3 (ICC 200; ERBE Corp., Tuebingen, Germany) or ESG-100 in 50-Watt pulse cut slow mode (Olympus Corp., Tokyo, Japan). Endoscopic papillary large balloon dilatation (EPLBD) was defined as papillary dilatation performed using a balloon of ≥12-mm diameter, and endoscopic papillary balloon dilatation (EPBD) was defined as papillary dilatation performed using a balloon of ≤10-mm diameter. EPLBD was performed for the papilla after performing EST or with a history of EST. Endoscopic nasobiliary drainage (ENBD) was performed using a 6-Fr catheter, and endoscopic biliary drainage (EBD) was performed using a 7-Fr stent. Complete stone removal confirmation was performed by cholangiography using balloon catheter. The contrast was injected from the proximal side hole of the balloon catheter after performing procedure for papilla such as EST. All patients were given antibiotics, with the type of antibiotic and administration period determined at the discretion of the attending physician. With the aim of preventing post-ERCP pancreatitis, on the day of the ERCP procedure, all patients were given 600 mg/day of gabexate mesilate. Furthermore, all patients underwent blood tests 3 h after the procedure and on the following day. Thereafter, blood and imaging tests were performed as required at the discretion of the attending physician to ascertain the patient's condition.

### 2.2. Measurements

In this study, the primary outcome was to evaluate the safety of early stone removal for mild-to-moderate acute cholangitis associated with choledocholithiasis, and the secondary outcome was to evaluate the clinical benefit of early single-session stone removal.

We retrospectively compared the patient characteristics, the details of ERCP procedure, the procedural duration, total number of ERCP sessions, stone removal rate, cholangitis improvement rate, period from early ERCP to the second ERCP for stone removal, length of hospital stay, and procedural complications in patients in the removal group (n = 78) with those in the drainage group (n=89). Furthermore, procedural complications were compared between patients who underwent EST in early ERCP and those who underwent EST in elective ERCP following improvement in cholangitis in the drainage group. Moreover, stepwise multiple linear regression analysis was performed to evaluate the factors related to hospitalization period.

Improvement of cholangitis was defined as a body temperature <37°C maintained for >24h and normalization of white blood cell count in blood test. On the other hand, worsening of cholangitis was defined as that blood test values and/or clinical symptoms such as fever or abdominal pain showed an exacerbation tendency. The diagnosis of procedural complications included pancreatitis, perforation, and cholangitis based on the consensus guidelines by Cotton et al. [12]. Post-EST bleeding was defined as requiring endoscopic hemostasis. However, simple hemostasis with coagulation using EST knife immediately after EST was excluded.

The present study was performed with the approval of the ethical review board of our hospital (approval number: 3667). Informed consent about present study participation was officially announced on a web page.

### 2.3. Statistical Analysis

Chi-square test, Fisher's exact test, and Welch's *t*-test were used for statistical analysis where appropriate. Stepwise multiple linear regression analysis with a forward approach using cutoff value of 0.05 was performed to identify independent predictors of hospitalization period. A P value of <0.05 was regarded as significant. Statistical analysis was performed with StatMate IV (ATMS Co., Ltd., Tokyo, Japan) and SPSS (version 19; SPSS, Chicago, IL, USA).

## 3. Results

### 3.1. Patient Characteristics

Patient characteristics are shown in Table 1. There was no difference in terms of gender, performance status (PS), severity of cholangitis, proportion of post-EST papilla, and presence or absence of parapapillary diverticulum between the removal and drainage groups; however, the patients in the removal group were significantly younger and had fewer and smaller stones compared with those in the drainage group. Moreover, the drainage group included many patients who were taking antithrombotic agents.

### 3.2. Procedures

The details of the early ERCP procedure are shown in Table 2. Biliary cannulation was achieved in all 167 patients. Techniques for successful biliary cannulation included CC in 69 patient, P-GW in 7, and Pre-cut in 2 in the removal group and CC in 77 and P-GW in 12 in the drainage group. There was no difference in the technique of successful biliary cannulation between the two groups. The procedures for papilla included EST in 58 patients, EPBD in 9, and EPLBD in 8 in the removal group and EST in 11 in the drainage group. EST, EPBD, EPLBD, and intraductal ultrasonography were more frequently performed in the removal group, whereas endoscopic biliary stenting (EBS) was more frequently performed in the drainage group. There was no difference in the proportion of pancreatic stenting between the two groups (P = 0.229).

In 84 out of the 89 patients in the drainage group, elective stone removal was performed after cholangitis had improved. The procedures for papilla involved EST in 37 patients, EPBD in 13, EPLBD in 26, and pre-cutting in 1. Furthermore, five patients had concurrent malignant tumors or severe illness and were thus discharged after undergoing EBD alone without stone removal.

### 3.3. Therapeutic Outcomes

The therapeutic outcomes are shown in Table 3. The procedural duration for early ERCP was 38.8 min in the removal group and 27.0 min in the drainage group, which was significantly shorter (P = 0.036); however, the total number of ERCP sessions was 1.2 in the removal group and 2.2 in the drainage group, indicating that significantly fewer sessions were performed for the removal group (P < 0.001). The single-session complete stone removal rate in early ERCP was 85.9% for the removal group. The overall complete stone removal rate was 100% for the removal group and 94.4% for the drainage group, with no significant difference observed (P = 0.095). The improvement rate in acute cholangitis following early ERCP and the overall improvement rate in acute cholangitis were 100% for both groups. The mean period from early ERCP to second ERCP for stone removal was 7.8 days for the removal group and 9.8 days for drainage group with no statistical difference (P = 0.068). The mean length of hospital stay was 11.9 days for the removal group and 19.9 days for the drainage group, indicating a significantly shorter stay for the removal group (P < 0.001).

### 3.4. Procedural Complications

The procedural complications in early ERCP are shown in Table 4. The overall procedural complication rate was 11.5% for the removal group and 10.1% for the drainage group, with no significant difference observed between the two groups (P = 0.963). In the removal group, pancreatitis occurred in 2.6%, post-EST bleeding in 5.1%, post-EPLBD bleeding in 1.3%, Mallory–Weiss syndrome in 1.3%, and ENBD self-removal in 1.3%, whereas in the drainage group, pancreatitis occurred in 1.1%, post-EST bleeding in 1.1%, Mallory–Weiss syndrome in 1.1%, ENBD self-removal in 3.4%, and stent migration in 3.4%; no difference was observed between the two groups. Furthermore, perforation, worsening of cholangitis, and acute cholecystitis were not observed in either group, and there were no fatalities. In patients who underwent early EST, including those in the removal and drainage groups, the post-EST bleeding rate was 6.8% (5/69), including 6.9% (4/58) in the removal group and 9.1% (1/11) in the drainage group, with no significant difference observed (P = 0.706). Moreover, upon comparing early EST (n = 69) with elective EST after cholangitis had improved (n = 37), the post-EST bleeding rates were 6.8% (5/69) and 2.7% (1/37), respectively, with no significant difference observed (P = 0.600) (Table 5). In all patients with post-EST bleeding and post-EPLBD bleeding, hemostasis was achieved by endoscopic hemostasis (injection of hypertonic saline-epinephrine: 4, compression using balloon catheter for stone removal or EPBD: 3, and clipping: 1), and all other complications were improved with conservative treatment.

### 3.5. Predictors of Hospitalization Period

Stepwise multiple linear regression was calculated to predict hospitalization period. Independent variables included age, sex, PS, severity of cholangitis, white blood cell count (WBC), C-reactive protein (CRP) level, total bilirubin (T-Bil) level, number of stones, diameter of stones, naïve papilla, use of antithrombotic drug, procedure for naïve papilla in early ERCP, stone removal in early ERCP, development of adverse event, development of pancreatitis, and development of bleeding. A significant regression equation was found (F (3, 163) = 18.973, p < 0.001, with an R^2^ of 0.259). Participants' predicted hospitalization period is equal to 9.008 + 0.250 (CRP level) + 0.568 (number of stones) + 7.467 (stone removal in early ERCP), where CRP level is measured in mg/dl, and stone removal in early ERCP is coded as 1 = without, 0 = with. Participants' hospitalization period increased 7.467 days in without stone removal in early ERCP, 0.568 days for each number of stones, and 0.250 days for each mg/dl of CRP level. As a result, stone removal in early ERCP, number of stones, and CRP level were significant predictors of hospitalization period (Table 6).

## 4. Discussion

In this study, we evaluated the safety and clinical benefit of single-session stone removal for mild-to-moderate acute cholangitis associated with choledocholithiasis. As a result, regarding safety, there was no difference in the incidence of adverse events such as cholangitis exacerbation or post-EST bleeding between single-session stone removal and only drainage; that is, it was considered that the single-session stone removal could be performed with the same safety level as only drainage. Regarding clinical benefit, single-session stone removal contributed to a significant shortening of the hospitalization period. Furthermore, a multiple linear regression analysis revealed that the factor most related to the hospitalization period was stone removal in early ERCP. Therefore, in this study, we found that single-session stone removal for mild-to-moderate acute cholangitis can be performed safely, moreover, with the perspective of shorter length of hospital stay.

As for EST in the presence of acute cholangitis, Sugiyama et al. [6] retrospectively compared the presence or absence of EST prior to ENBD for acute cholangitis and found that, in patients without EST, the overall complication rate was 2% and the bleeding rate was 0%, whereas in those with EST, the overall complication rate was 11% and the bleeding rate was 4%, which were significantly higher (P < 0.05). However, in the report, among patients who underwent EST, 19% had severe cholangitis and 8% had coagulopathy. In addition, two among the three patients with bleeding had coagulopathy. Hui et al. [7] retrospectively compared the presence or absence of EST prior to EBS for acute cholangitis associated with choledocholithiasis. The overall complication rate was 2.7% and the bleeding rate was 0% in patients without EST, whereas the overall complication rate was 10.8% and the bleeding rate was 8.1% in patients with EST, indicating higher rates in those who underwent EST. However, in the report by Hui et al. [7], all patients had severe cholangitis or acute suppurative cholangitis. Therefore, for patients with severe acute cholangitis, EST may increase the risk of bleeding.

On the other hand, Ueki et al. [9] retrospectively compared single-session EST against elective EST prior to EBD for moderate acute cholangitis associated with choledocholithiasis and reported that the rate of bleeding was 13% in single-session EST and 5% in elective EST, with no significant difference observed (P = 0.127). In addition, Eto et al. [10] reported that the rate of post-EST bleeding of single stage EST for mild-to-moderate cholangitis was 4% (2/5), and Ito et al. [11] reported that the rate of post-EST bleeding of immediate EST for acute suppurative cholangitis was 0% (0/59). Therefore, these reports concluded that the risk of post-EST bleeding is not high in patients with mild-to-moderate cholangitis. In the present study, the post-EST bleeding rate among patients with EST was 6.8% (removal group: 6.9%, drainage group: 9.1%), which was comparable to that in previously reported results, and on comparing early EST with elective EST after cholangitis had improved, there was no difference in procedural complications including bleeding; thus, it was suggested that, in mild-to-moderate cholangitis, EST can be performed with equal safety similar to that performed in patients without cholangitis.

With regard to single-session stone removal, Jang et al. [13] retrospectively compared urgent and elective single-session stone removal for mild-to-moderate acute cholangitis and demonstrated that there was no significant difference in clinical success rate and intervention-related complications. Furthermore, the urgent group has a shorter length of hospital stay (P < 0.001). Moreover, Eto et al. [10] conducted a prospective single-arm study of single-session stone removal for 50 patients with mild-to-moderate acute cholangitis associated with choledocholithiasis and reported that, within 4 days of the procedure, the improvement rate of acute cholangitis was 90%, the overall procedural complication rate was 10%, and the bleeding rate was 4%; thus, in the absence of coagulopathy and oral antithrombotic therapy, they found that single-session stone removal by EST was useful. In the present study, we observed no cases of cholangitis exacerbation following single-session stone removal, with an improvement observed in cholangitis in all cases. Therefore, if the acute cholangitis is mild-to-moderate, then we consider that single-session stone removal does not carry a high risk of cholangitis exacerbation. Furthermore, other procedural complication rate, such as pancreatitis, was comparable in patients who underwent drainage alone; thus, single-session stone removal by EST can be safely performed.

Same as present study, Ito et al. [11] compared the therapeutic results of single-session stone removal for acute cholangitis associated with choledocholithiasis with those of elective stone removal following drainage and reported that there was no difference in the cholangitis improvement rate and procedural complication rate. However, it cannot be ruled out that the results were affected by the fact that the sample size for elective stone removal following drainage was small at 28 patients and that the single-session complete stone removal rate was low at 30.5%. The present study included more patients (removal group: 78 patients, drainage group: 89 patients), and the single-session complete stone removal rate was high at 85.9%. However, consequently, there was no difference in the cholangitis improvement rate and procedural complication rate, which is consistent with the findings by Ito et al. Therefore, even if stone removal was performed with a high complete stone removal rate, it could be safely performed without exacerbation of cholangitis. Furthermore, in the report by Ito et al., there was no statistically significant difference in the length of hospital stay between the single-session stone removal group (n=59) and the group that underwent elective stone removal after drainage (n=28) (P=0.09). However, when limited to the patients with successful complete stone removal in the single-session stone removal group (n=18), the hospitalization period was significantly shorter than the elective EST group (P=0.02). In the present study, the removal group had a significantly shorter length of hospital stay and we consider that the high complete stone removal rate in the removal group could have contributed to shorter length of hospital stay. Since stone removal in early ERCP was identified as a factor most related to hospitalization period in multiple linear regression analysis, high complete stone removal rate may contribute to shortening of hospitalization period. Besides, the number of stones and CRP level are also identified as a factor related to hospitalization period; therefore, requiring multiple ERCP sessions for many stones or severe inflammation may have affected the prolonged hospitalization.

Limitations of the present study are that it was a retrospective study and that there may have been a selection bias in the drainage group comprising patients receiving oral antithrombotic therapy, patients with many stones, and patients with large stone. Therefore, further prospective comparative studies of patients with consistent backgrounds are needed.

## 5. Conclusions

In the present study, we found that, in patients with mild-to-moderate acute cholangitis associated with choledocholithiasis, without receiving oral antithrombotic therapy, single-session stone removal can be performed with the same safety level as that for elective stone removal after drainage, moreover, with the perspective of shorter length of hospital stay.

## Figures and Tables

**Table 1:** Patient characteristics.

	Removal group (n=78)	Drainage group (n=89)	*P* value
Age (mean±SD)	71.1±16.1	77.3±9.9	0.002
Sex (Male/Female)	41/37	52/37	0.447
Performance status (0-2/3-4)	66/12	72/17	0.527
Severity of cholangitis (Moderate/Mild)	37/41	39/50	0.640
Blood test values			
WBC (/*μ*l) (mean±SD)	10661±4280	10903±4320	0.717
CRP (mg/dl) (mean±SD)	6.26±5.55	6.09±6.09	0.851
T-Bil (mg/dl) (mean±SD)	3.80±2.71	3.30±2.42	0.213
Number of stones (mean±SD)	2.33±2.29	3.37±3.47	0.002
Diameter of stones (mm)(mean±SD)	8.55±4.80	12.24±6.36	<0.001
Post-endoscopic sphincterotomy (n (%))	19 (24.4)	23 (25.8)	0.825
Parapapillary diverticulum (n (%))	44 (56.4)	52 (58.4)	0.793
Antithrombotic drug (n (%))	6 (7.7)	59 (66.3)	<0.001

SD: standard deviation, WBC: white blood cell count, CRP: C-reactive protein, and T-Bil: total bilirubin.

**Table 2:** Procedures of early endoscopic retrograde cholangiopancreatography.

Endoscopic procedures (n (%))	Removal group (n=78)	Drainage group (n=89)	*P* value
Techniques for successful biliary cannulation			
CC	69 (88.5)	77 (86.5)	0.705
P-GW	7 (9.0)	12 (13.5)	0.360
Pre-cut	2 (2.6)	0 (0)	0.420
Endoscopic sphincterotomy	58 (74.4)	11 (12.4)	<0.001
Endoscopic papillary balloon dilatation	9 (11.5)	0 (0)	0.003
Endoscopic papillary large balloon dilatation	8 (10.3)	0 (0)	0.006
Intraductal ultrasonography	8 (10.3)	1 (1.1)	0.024
Endoscopic mechanical lithotripsy	10 (12.8)	–	–
Basket catheter	42 (53.8)	–	–
Balloon catheter	49 (62.8)	–	–
Endoscopic nasobiliary drainage	9 (11.5)	16 (18.0)	0.245
Endoscopic biliary stenting	8 (10.3)	74 (83.1)	<0.001
Incidental pancreatography	30 (38.5)	33 (37.1)	0.854
Pancreatic stenting to prevent pancreatitis	6 (7.7)	12 (13.5)	0.229

CC: conventional contrast cannulation, P-GW: pancreatic duct guidewire placement method.

**Table 3:** Comparison of the treatment results between the removal and drainage groups.

	Removal group (n=78)	Drainage group (n=89)	*P* value
Procedure time of early ERCP (min)(mean±SD)	38.8±18.6	27.0±15.2	0.036
Number of ERCP administrations (mean±SD)	1.2±0.50	2.2±0.8	<0.001
Complete stone removal ratio in early ERCP (n (%))	67 (85.9)	–	–
Final complete stone removal ratio (n (%))	78 (100)	84 (94.4)	0.095
Improvement rate of cholangitis after early ERCP (n (%))	78 (100)	89 (100)	–
Final improvement rate of cholangitis (n (%))	78 (100)	89 (100)	–
Period to second ERCP for stone removal (day)(mean±SD)	7.8±3.9	9.8±4.7	0.068
Hospitalization period (day)(mean±SD)	11.9±7.16	19.9±8.69	<0.001

ERCP: endoscopic retrograde cholangiopancreatography, SD: standard deviation.

**Table 4:** Comparison of adverse events in early ERCP between the removal and drainage groups.

	Removal group (n=78)	Drainage group (n=89)	*P* value
Adverse events ratio (n (%))	9 (11.5)	9 (10.1)	0.963
Pancreatitis	2 (2.6)	1 (1.1)	0.484
Post-EST bleeding	4 (5.1)	1 (1.1)	0.259
Post-EPLBD bleeding	1 (1.3)	0 (0)	0.947
Perforation	0 (0)	0 (0)	–
Worsening cholangitis	0 (0)	0 (0)	–
Acute cholecystitis	0 (0)	0 (0)	–
Mallory-Weiss syndrome	1 (1.3)	1 (1.1)	0.536
ENBD self-removal	1 (1.3)	3 (3.4)	0.709
Stent migration	0 (0)	3 (3.4)	0.292

EST: endoscopic sphincterotomy, EPLBD: endoscopic papillary large balloon dilatation, and ENBD: endoscopic nasobiliary drainage.

**Table 5:** Comparison of adverse events between early and elective endoscopic sphincterotomy.

	Early EST (n=69)	Elective EST (n=37)	*P* value
Adverse events ratio (n (%))	7 (10.1)	1 (2.7)	0.319
Pancreatitis	2 (2.9)	0 (0)	0.767
Post-EST bleeding	5 (6.8)	1 (2.7)	0.600
Perforation	0 (0)	0 (0)	–
Worsening cholangitis	0 (0)	0 (0)	–
Acute cholecystitis	0 (0)	0 (0)	–

EST: endoscopic sphincterotomy.

**Table 6:** Stepwise multiple linear regression analysis for variables associated with hospitalization period.

Variables	B	SE	*β*	T value	P value
Stone removal in early ERCP	7.467	1.223	0.418	6.107	< 0.001
Number of stones	0.568	0.203	0.192	2.792	0.006
CRP level	0.250	0.104	0.163	2.412	0.017

B: partial regression coefficient, SE: standard error, *β*: standardized partial regression coefficient, ERCP: endoscopic retrograde cholangiopancreatography, and CRP: C-reactive protein.

## Data Availability

The datasets generated and/or analyzed during the current study are available from the corresponding author on reasonable request.
